# Probabilistic forecasting of maximum human lifespan by 2100 using Bayesian population projections

**DOI:** 10.4054/demres.2021.44.52

**Published:** 2021-06-30

**Authors:** Michael Pearce, Adrian Raftery

**Affiliations:** 1 Departments of Statistics, University of Washington, USA.; 2 Departments of Statistics and Sociology, University of Washington, USA.

## Abstract

**BACKGROUND:**

We consider the problem of quantifying the human lifespan using a statistical approach that probabilistically forecasts the maximum reported age at death (MRAD) through 2100.

**OBJECTIVE:**

We seek to quantify the probability that any person attains various extreme ages, such as those above 120, by the year 2100.

**METHODS:**

We use the exponential survival model for supercentenarians (people over age 110) of Rootzén and Zholud (2017) but extend the forecasting window, quantify population uncertainty using Bayesian population projections, and incorporate the most recent data from the International Database on Longevity (IDL) to obtain unconditional estimates of the distribution of MRAD this century in a fully Bayesian analysis.

**RESULTS:**

We find that the exponential survival model for supercentenarians is consistent with the most recent IDL data and that projections of the population aged 110–114 through 2080 are sensible. We integrate over the posterior distributions of the exponential model parameter and uncertainty in the supercentenarian population projections to estimate an unconditional distribution of MRAD by 2100.

**CONCLUSIONS:**

Based on the Bayesian analysis, there is a greater than 99% probability that the current MRAD of 122 will be broken by 2100. We estimate the probabilities that a person lives to at least age 126,128, or 130 this century, as 89%, 44%, and 13%, respectively.

**CONTRIBUTION:**

We have updated the supercentenarian survival model of Rootzén and Zholud using the most recent IDL data, incorporated Bayesian population projections, and extended the forecasting window to create the first fully Bayesian and unconditional probabilistic projection of MRAD by 2100.

## 1. Introduction

Understanding longevity is of great importance, as it has big implications for government programs, economic policy, and individual planning. Although longevity has been studied scientifically for well over a century, the topic of human lifespan, quantified by the maximum reported age at death (MRAD) statistic, has been intensively investigated only since the 1990s (Marck et al. 2017).

Many authors have forecast an immutable, fixed limit to human lifespan. Some have argued that the flattening gains in life expectancy at birth that have been observed in many populations imply that MRAD will also fail to increase substantially in the coming decades (Olshansky, Carnes, and Cassel 1990; Dong, Milholland, and Vijg 2016). Similarly, many authors have suggested that strong biological forces limit human lifespan, such as the inevitable deterioration of cells that cannot be overcome, even as diseases like cancer, diabetes, or Parkinson’s continue to be addressed (Olshansky, Carnes, and Désesquelles 2001; Carnes and Olshansky 2007; Olshansky and Carnes 2009; Le Bourg 2012; Vijg and Le Bourg 2017). These conclusions have support in the biological literature as well. Hayflick (2007) argued that aging is not an addressable disease, but the result of “random losses in molecular fidelity.” Also, Steenstrup et al. (2017) found that the shortening of leukocyte telomeres below a certain threshold with age, a process that is “highly heritable and largely determined at birth,” strongly predicts imminent death.

Other authors have disagreed. Vaupel (1997) noted that mortality for people in their 80s and 90s has decreased significantly in recent decades, suggesting that old-age mortality is far more plastic than previously thought. Oeppen and Vaupel (2002) noted that between 1928 and 1990 proposed caps to human lifespan were always broken quickly, an average of five years after they had been proposed. They found that the flattening of life expectancy “is an artifact of laggards catching up and leaders falling behind,” not that it is somehow tied to caps on human lifespan. Responding to the criticism that the current record for lifespan (122 years and 164 days set by Jeanne Calment of France) has not changed since 1997, Gavrilov, Krut’ko, and Gavrilova (2017) noted that other periods have seen no major gains in MRAD or life expectancy, only to be followed by periods of dramatic improvement.

Also, biologists and researchers have recently found promising pathways to stop biological aging, such as in emerging drugs and therapies currently undergoing clinical trials (Ben-Haim et al. 2017; Flatt and Partridge 2018; Campisi et al. 2019; Pignolo 2019). Regarding the lack of evolutionary pressure past reproductive years, Kirkwood (2017) observed that lifespans may continue to increase as evolution still encourages health through youth and adulthood, which is increasingly seen as correlated with decreased mortality in senescence.

Perhaps the most unifying aspect of the debate at hand is its uncertainty. Researchers have found that the small number of people to have verifiably reached age 110, unknown future scientific breakthroughs, and lack of biological knowledge regarding the mechanisms of aging limit our ability to make definitive claims about limits to human lifespan (Vaupel 1997; Wilmoth 2000; Le Bourg and Vijg 2017; Robine and Cubaynes 2017). Despite arguing for the existence of a cap to human lifespan, Olshansky and Carnes (2009) agreed that the probability of survival at any given age cannot be exactly zero, leaving open the possibility of MRAD records being broken continuously as the centenarian population increases. They later noted that purely mathematical models cannot be used exclusively to predict MRAD, citing Zeno’s arrow paradox (Olshansky and Carnes 2019).

Despite substantial research, before 2010 statistical analyses of supercentenarians (those who live to at least age 110) were plagued by *age-attainment bias*, which is defined as the tendency of advanced-age people to exaggerate or round up their age (Poulain 2010). This problem was mitigated by the publication of the International Database on Longevity (IDL) by the Max Planck Institute for Demographic Research. At the time of its release, the IDL was the first unified dataset with rigorously verified birth, life, and death records of supercentenarians, including only those who age could be confirmed with a high degree of certainty (Poulain 2010).

Since 2010, a number of analyses have employed the IDL data to study the possibility of a limit to human lifespan. Notably, Dong, Milholland, and Vijg (2016) proposed an immutable cap to human lifespan at age 115, barring outliers such as Jeanne Calment. Although this paper was roundly criticized for methodological issues (Lenart and Vaupel 2017; Rozing, Kirkwood, and Westendorp 2017; Hughes and Hekimi 2017), its principal arguments have also been modified to support a cap to human lifespan at age 125 (de Beer, Bardoutsos, and Janssen 2017).

In this paper, we reexamine and extend the rebuttal of Dong, Milholland, and Vijg by Rootzén and Zholud (2017) (henceforth referred to as RZ) in support of the “human life is unlimited, but short” hypothesis. This hypothesis builds upon the evidence that mortality beyond age 110 does not follow an increasing Gompertzian pattern, but instead plateaus by age 110 at approximately 50% year-over-year mortality (see Robine and Vaupel 2002; Gampe 2010; later supported by Barbi et al. 2018; Feehan 2018; Belzile et al. 2020). Although the flattening of the mortality curve for supercentenarians may appear implausible, research suggests that these lucky individuals simply do not follow the mortality patterns of most people: For example, da Silva Antero-Jacquemin et al. (2015) found that Olympic athletes, presumably some of the world’s healthiest individuals, experience mortality similar to the world population at large.

The hypothesized constant survival probability past age 110 suggests an exponential supercentenarian population model, which embodies the idea that human life is theoretically unbounded but unlikely to extend well beyond currently observed levels. The key contribution of RZ was to estimate a density curve of the maximum human lifespan by 2042 based on their model, using order statistics and a nonprobabilistic projection of the number of people to reach age 110 between 2018 and 2042.

This paper extends the work of RZ in three ways: (1) by incorporating Bayesian population projections into a probabilistic and unconditional posterior density for maximum human lifespan, (2) by extending the projections to 2100, and (3) by using updated data from the IDL.

The paper is organized as follows. In Section 2 we review the exponential survival model for supercentenarians and present updated estimates of the model for the IDL version 3 data. In Section 3 we describe our methodology, and give results in Section 4. We conclude with a discussion in Section 5.

## 2. The exponential survival model for supercentenarians

### 2.1 Model

RZ tested a variety of supercentenarian survival models using statistical Extreme Value Theory. Ultimately, they found that a simple, exponential survival model best fit the data. The exponential survival model beyond age 110 is notable for a few reasons. First, it is a single parameter distribution that does not incorporate any covariates, which suggests that survival probabilities are equivalent beyond age 110 regardless of sex, nationality, or genetic background. Also, the exponential survival model suggests that the probability of surviving one additional year conditional on current age is constant, due to the memory-less property of the exponential distribution. It is worth noting that this model does not suggest that equal numbers of people by sex, nationality, or genetic background will attain supercentenarian ages, nor that populations are constant beyond age 110. Instead, it only implies that *conditional* on attaining any age past 110, the probability of surviving one additional year is equal regardless of any other characteristic.

We now describe the model. Let X be a random variable that represents the age at death minus 110. Then,

(1) $$X\sim \text{Exponential}\left(\lambda \right),$$

such that $E[X]=\frac{1}{\lambda }$ and X has probability density function at age x equal to $\lambda e^{-\lambda x}$. Then the probability of surviving one additional year, conditional on attaining some fixed age x at or beyond 110, is s1x=e−λ, which is independent of x.

Now, suppose we have an independent and identically distributed sample of N people who attain age 110, and let $X_{1},\ldots ,X_{N}$ be the ages at which they die, minus 110. Let $X_{\left(N\right)}={\text{max}}_{i=1,\ldots ,N}X_{i}$ be the age at death of the person who survives longest past age 110, also called the maximum order statistic of the ages at death. Then the probability density function of $X_{\left(N\right)}$ is

(2) $$f_{\left(N\right)}(x|\lambda )=N\lambda e^{-\lambda x}{\left[1-e^{-\lambda x}\right]}^{N-1}$$

(De Haan and Ferreira 2007).

RZ obtained a point estimate and confidence interval for λ using maximum likelihood estimation that incorporated the truncation and sampling patterns present in the IDL (see Section 2.2 for details). They then obtained a point estimate and confidence interval for the number of people to attain age 110 in the period 2018–2042, N, as follows. First they estimated the number of people to die in Italy, England and Wales, and the United States using Poisson regression with linear link function in the period 1980–1999. They then extrapolated to the period of interest and multiplied that estimate and confidence interval by the historical supercentenarian population ratio between those countries and all others represented in the IDL database. Ultimately, they estimated the mean of the exponential distribution, 1/λ, to be 1.34 with 95% confidence interval (1.22, 1.46) and N to be 2, 974 (2, 334, 3, 615). Plugging these values into equation (2) yields a density curve of maximum age at death in the period 2018–2042.

We now point out two issues with the analysis of RZ. First, the authors use a very simple estimation technique to find N, which relies only on a portion of the populations of interest and fails to adequately capture the uncertainty in the population estimates, especially since the number of people reaching advanced ages is expected to increase sharply over the next several decades due to fast population growth. Second, their projection period covers only 25 years after publication, perhaps due to unwillingness to extrapolate far beyond the population model’s training data. These issues will be resolved by the fully Bayesian method presented in Section 3. But first, we confirm the exponential survival model and update RZ’s analysis using data in the most recent version of the IDL.

### 2.2 Application to IDL version 3 data

Since the original publication of the IDL in 2010, there have been two updates. The current version (“IDL v3”; described in Jdanov, Shkolnikov, and Gellers-Barkmann 2021) contains n=1,119 records of deceased individuals who attained at least age 110 and represent 13 countries: Austria, Belgium, Canada, Denmark, England and Wales, Finland, France, Germany, Japan, Norway, Spain, Sweden, and the United States, which we will refer to as the “IDL countries.”^3^ In this collection, the five most represented countries are the United States (504 observations), France (241), England and Wales (157), Japan (78), and Spain (60). We notice that the number of supercentenarians is roughly proportional to their comparative population sizes. A complete set of overall and sex-specific counts for the IDL countries can be found in the Appendix Table A-1.

IDL v3 improved upon previous versions by adding additional supercentenarian records from existing countries and one additional country (Austria), and by removing data from Australia that may have exhibited age-attainment bias. Unfortunately, there were also records removed from the IDL due to new privacy regulations, specifically for supercentenarians from Switzerland and Italy (Jdanov, Shkolnikov, and Gellers-Barkmann 2021; Max Planck Institute for Demographic Research 2020).

The IDL v3 also includes the records of 13,931 individuals who died between the ages of 105–109, called *semisupercentenarians*, who come from 9 of the 13 IDL countries (there were no entries from Finland, Japan, Spain, or Sweden). The semisupercentenarian records were excluded from the analysis due to statistically significant evidence suggesting a difference in mortality between semisupercentenarians and supercentenarians, as well as sex- and region-specific differences in mortality for the former group. See Appendix for details.

**Figure 1:**
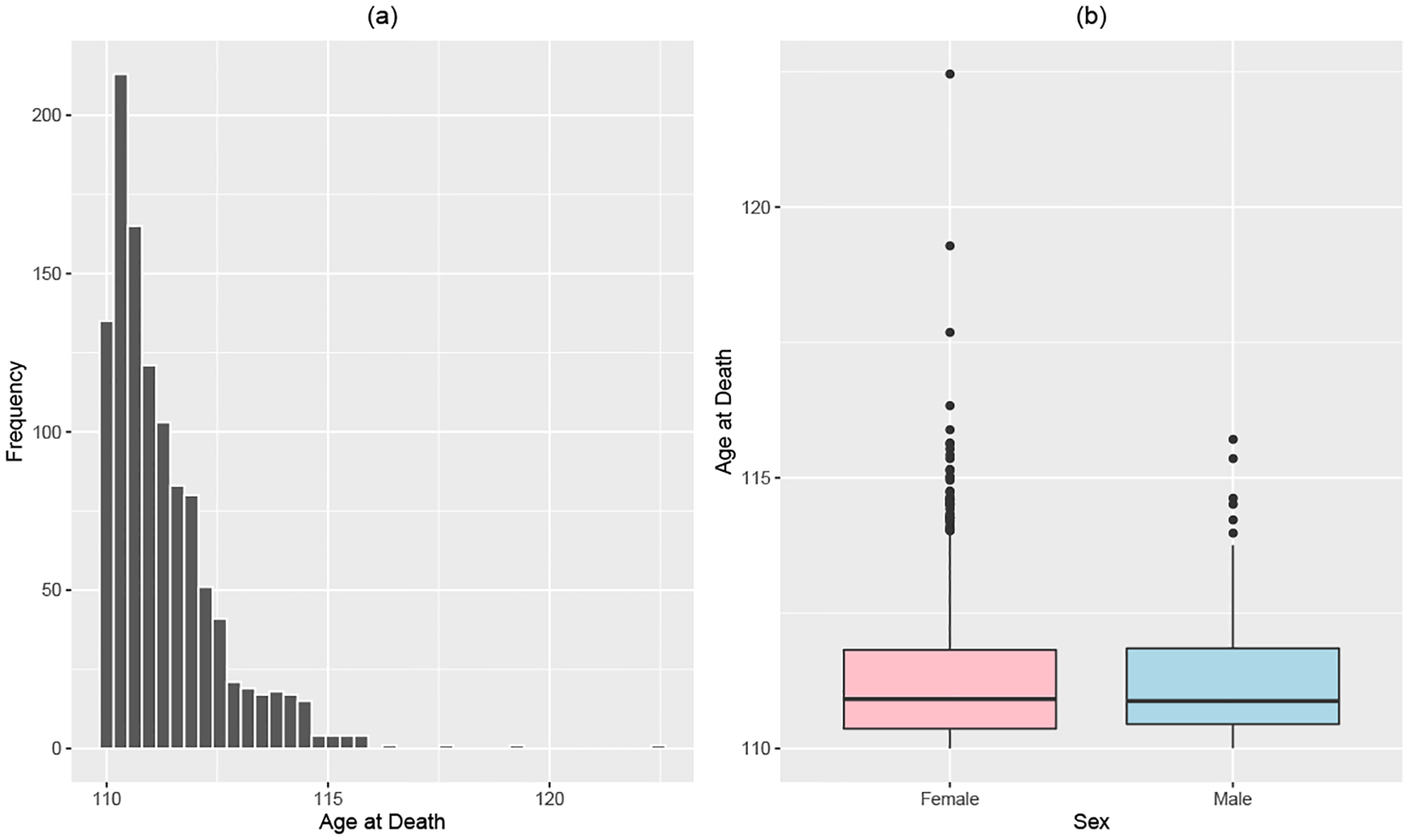
Histogram of age at death (a), side-by-side boxplots of age at death by sex (b)

Before fitting an exponential model to the data, we perform exploratory analyses and test model assumptions. Figure 1 displays histograms and boxplots of age at death, both overall and by sex. We notice the exponential decay pattern to the survival data in the left panel, as well as the similar minimum, first quartile, median, third quartile, and maximum values of age at death between males and females in the right panel, which supports the exponential survival model. The number of high outliers for females is likely due to the sheer size of the female group (1,029 observations compared to 90 observations for males).

**Figure 2:**
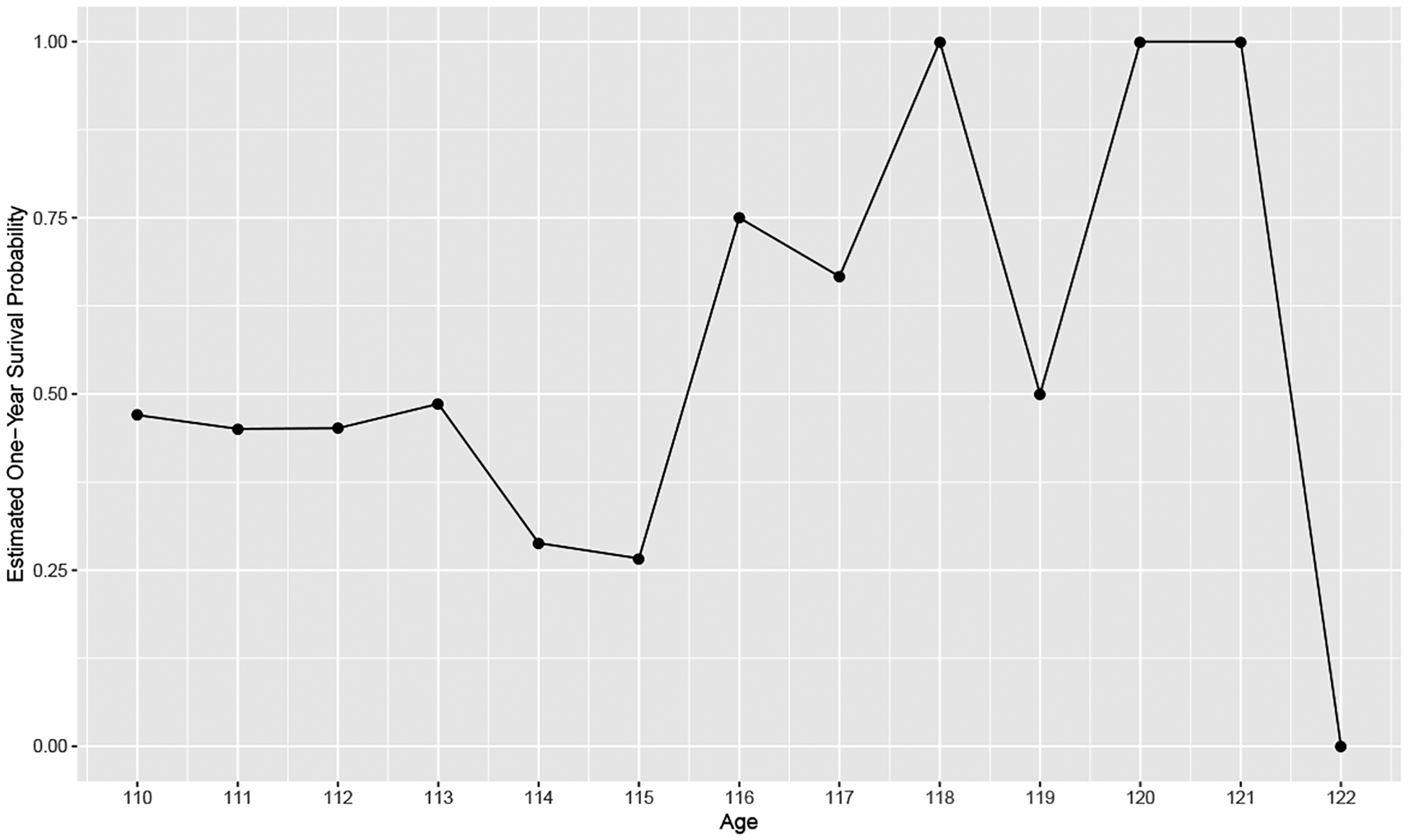
Estimated one-year survival probabilities for supercentenarians from the IDL v3 data

We also examine the one-year survival probabilities, s1x, for x=110,111,…,122 in Figure 2. For ages 110–113, the survival probabilities look relatively flat. The volatility in s1x beyond age 113 can be attributed to the small sample size used when calculating those empirical probabilities. Therefore, the constant one-year survival probability implied by the model still seems reasonable.

At this point, it is relevant to state the IDL’s data sampling patterns. For each IDL country c, individuals are eligible for inclusion in the database only if they died at or beyond age 110 within a country-specific time interval, $\left(b_{c},e_{c}\right)$. This introduces truncation bias into the observations that are included in the IDL since birth cohorts are not followed to extinction and the probability of attaining age 110 may vary over time (Jdanov, Shkolnikov, and Gellers-Barkmann 2021).

We now describe how we handle the truncation bias present in the IDL. Suppose we observe a sample of individuals i=1,…,n, each from some country $c_{i}$. Let $x_{i}$ be the age at death for individual i minus 110 (in years), and let $t_{i}$ be the time at which the individual reached age 110. Then, let f and F be the probability and cumulative density functions, respectively, for the distribution of age at death minus 110. For individuals in the IDL, there are two cases to consider: (1) $t_{i}\leq b_{c_{i}}$ and (2) $t_{i}>b_{c_{i}}$.

In the first case, we only observe the individual if they lived long enough (but not too long) after attaining at 110 to die within the interval $\left(b_{c_{i}},e_{c_{i}}\right)$. This truncates the range of observable excess ages. Thus, the individual’s contribution to the joint likelihood should be

(3) $$\frac{f\left(x_{i}\right)}{F\left(e_{c_{i}}-t_{i}\right)-F\left(b_{c_{i}}-t_{i}\right)}.$$

In the second case, we only observe the individual if they do not live long enough to die after the year $e_{c_{i}}$. Thus, the individual’s contribution to the joint likelihood should be

(4) $$\frac{f\left(x_{i}\right)}{F\left(e_{c_{i}}-t_{i}\right)}.$$

In all subsequent statistical tests and model fitting, the sampling pattern described above is incorporated. Country-specific intervals $\left(b_{c},e_{c}\right)$ are determined based on the IDL metadata files.

Now, we formally test the model assumptions of flat mortality and no differences in mortality based on region or sex using likelihood ratio tests (LRT) and the Bayesian Information Criterion (BIC) as defined by Raftery (1995). For each of the tests described below, a large p-value or a positive BIC indicates lack of evidence against the null hypothesis model, and a small p-value or a negative BIC indicates evidence in favor of the alternative hypothesis model. Results are shown in Table 1.

To look for evidence of nonconstant mortality after age 110, we test the null hypothesis of an exponential survival model against the alternative of a Generalized-Pareto (GP) model with location parameter μ=0, which is a generalization of the exponential model by inclusion of an additional shape parameter to allow for constant mortality. Finding no evidence against the null hypothesis of an exponential survival model, we then test for differences in mortality based on region and sex, as well as sex-specific differences within regions. In each test, the null hypothesis is of a single-parameter exponential survival model (no differences in mortality based on sex or region), where the alternative hypothesis allows for differences in mortality based on sex or region.

We use four regions: North America (Canada and the United States), Northern Europe (Belgium, England and Wales, Denmark, Finland, Germany, Norway, and Sweden), Southern Europe (Austria, France, and Spain), and Japan. Testing is generally not performed at the country level due to small sample sizes. In each test, we find no statistically significant evidence to reject the null hypothesis. The single small p-value in Table 1 is likely due to supercentenarian data from Japan, which contains numerous individuals with deaths over age 115. Still, the p-value does not provide substantial evidence to reject the null hypothesis, especially in the presence of multiple testing. Therefore, we fail to find evidence against the simple exponential survival model for supercentenarians.

Next, we fit the exponential model to our data. Using maximum likelihood estimation and parametric bootstrap standard errors, we estimate the rate parameter of the exponential distribution to be $\overset{ˆ}{\lambda }=0.733$ with 95% confidence interval (0.689, 0.781). This corresponds to an estimated mean in the exponential distribution of 1.364 (1.280, 1.451). We emphasize that these frequentist results are stated only to illustrate their similarity with the results of RZ and confirm model fit; they are not used in our fully Bayesian estimation of the distribution of MRAD this century in Section 4.

**Figure 3:**
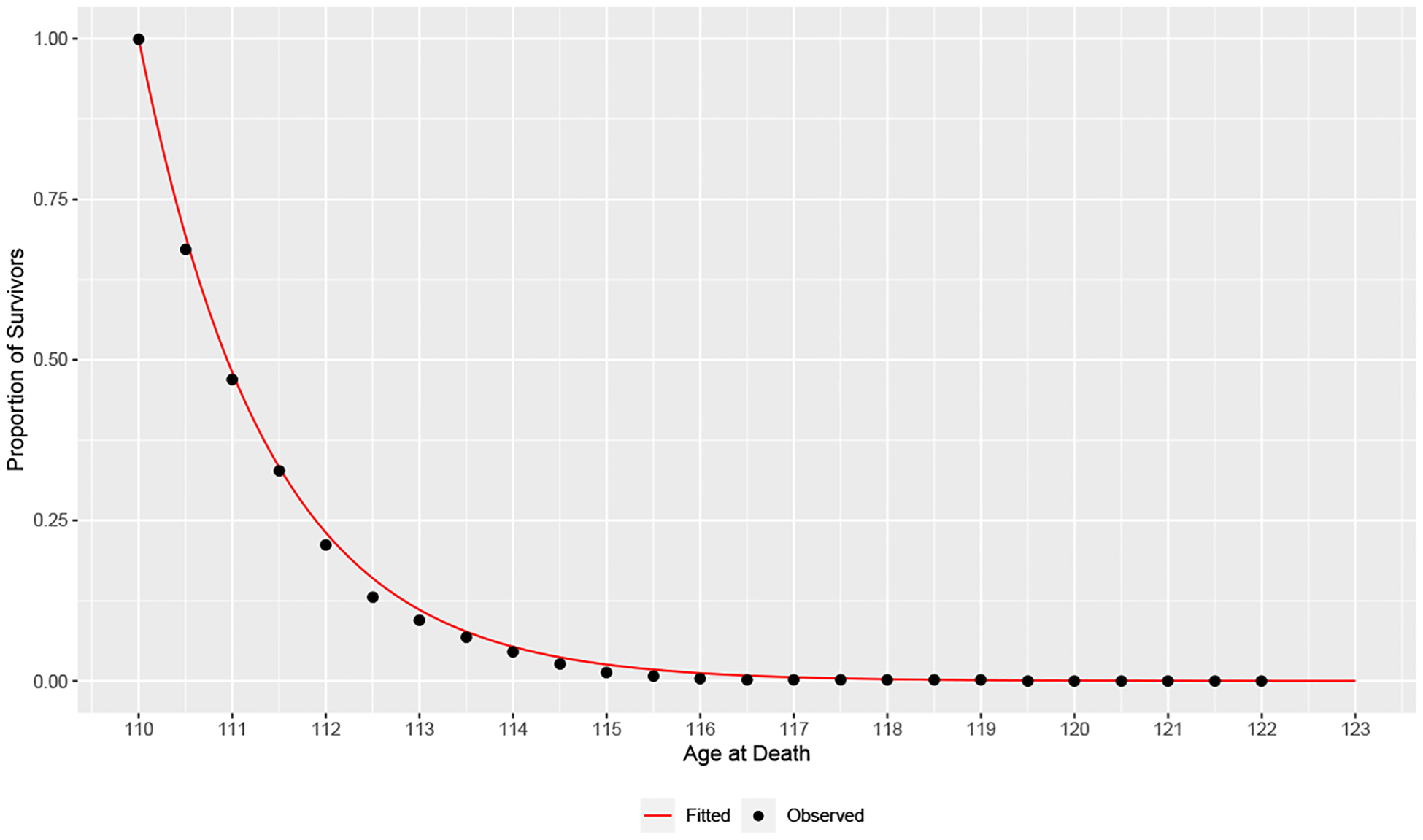
Fitted and observed survival proportions vs. age at death for supercentenarians from the IDL v3

To check for model parsimony, we overlay the proportion of observations in the dataset to attain each age in half-year increments by the fitted model values, appropriately scaled for comparison (Figure 3). Visual inspection suggests good model fit. We note that discrepancies may be attributed to normal variation given small sample sizes, as well as the data sampling patterns of the IDL.

We conclude by noting two minor concerns with the IDL v3 data. In recent years, there has been some debate in the literature regarding the veracity of Jeanne Calment’s record (Zak 2019; Robine et al. 2019). Although the evidence against Calment’s record is not strong, we ran our analyses with and without her record and found no substantially different results. A second concern regards the US data, which does not include a specific date of death for any supercentenarian records. For these individuals, we followed RZ and recorded their date of death as July 1 but note that results do not change substantially if they are instead recorded as January 1 or December 31.

## 3. Methodology

We now present the methodology of our Bayesian analysis in three steps. We will project the maximum reported age at death (MRAD) for individuals dying in the period 2020–2100 from any of the 13 IDL countries, as follows:

1. Confirm the single-parameter exponential survival model for supercentenarians and use order statistics to characterize the density of the MRAD this century conditional on the exponential parameter λ and the number of supercentenarians N this century, as described in Section 2.
2. Use Bayesian population projections to probabilistically forecast the number of people who may survive to age 110 this century.
3. Estimate the unconditional posterior distribution of MRAD this century using sampling from the posterior distributions of λ (using a vague prior) and of the supercentenarian population projections.

The first step was carried out in Section 2. We now describe the latter two steps.

### 3.1 Bayesian population projections

We create Bayesian population projections for the 13 IDL countries through 2080, which roughly allows those attaining age 110 in 2080 sufficient time to die before 2100 (under the assumption that MRAD will not extend far beyond age 130, which seems reasonable based on the previous results and those seen in Section 4).

Bayesian population projections are obtained using the method first discussed in Raftery et al. (2012). These projections take account of uncertainty regarding future levels of total fertility and life expectancy using Bayesian hierarchical models, as well as between-country correlation for fertility rates and between-sex correlation of life expectancy (Alkema et al. 2011; Raftery et al. 2013; Fosdick and Raftery 2014; Ševčíková et al. 2016). This method has been used by the United Nations World Population Prospects (WPP) since 2015 (Raftery, Alkema, and Gerland 2014).

We note that the projection method of Raftery et al. (2012) does not make specific assumptions about flat mortality after age 110. However, it does account for age-, sex-, and country-specific mortality patterns across all age ranges over time. Details of these assumptions can be found in Ševčíková et al. (2016), which also shows that many countries have flat or nearly-flat estimated mortality after age 110. Regardless, we find the flat mortality assumption reasonable due to our own testing in Section 2, as well as the wide body of literature to support this assumption as discussed in Section 1.

Specific projections are obtained through the R implementation of this method in the package *bayesPop* (Ševčíková and Raftery 2016). The package, however, only provides the number of people reaching 5-year age ranges at 5-year intervals, so we develop a method to estimate the total number of people to attain age 110 in each 5-year time period. This is a particularly important step when modeling supercentenarians because so many individuals who reach age 110 will die before they reach even age 111, significantly distorting the number of people alive in the age 110–114 age window from the number of people who attained age 110. The method is established through the following proposition.

Suppose we observe P people in a 5-year age range, (x,x+5), at a given time, t. If we assume that λ is the true exponential survival parameter and that people attained age x in uniform increments over the prior time period (t−5,t), then the number of people to attain age x over the period (t−5,t) is N=M×P, where

(5) $$M=\frac{5\lambda }{1-e^{-5\lambda }}.$$

Suppose N people will attain age 110 in the interval (t−5,t), spread uniformly across the time interval. Since the survival curve is dictated by the one-year survival probability s1x=e−λ, we use the rectangle method to attain an estimate of the number of people we should expect to observe at time t, denoted P, from the N original people:

$$P=\frac{N}{5}\int_{0}^{5}e^{-\lambda x}dx\;=\frac{N\left(1-e^{-5\lambda }\right)}{5\lambda }.$$

Therefore, we have

$$N=\frac{5\lambda P}{1-e^{-5\lambda }}\equiv M\times P$$

where $M=\frac{5\lambda }{1-e^{-5\lambda }}$, as desired.

### 3.2 Unconditional density curve of MRAD this century

To obtain an unconditional density curve of MRAD this century, f(x), we first consider the following expression based on the posterior distribution of λ given the IDL data, and the posterior distribution of supercentenarian population projections, P, given the WPP 2019 data (United Nations 2019) which is fed into the projection method:

(6) $$f\left(x\right)=\int \int f\left(x|P,\lambda \right)f\left(P|\text{WPP}\;2019\;\text{Data}\right)f\left(\lambda |\text{IDL}\;\text{Data}\right)dPd\lambda \;=\int \int \left[M_{\lambda }P\lambda e^{-\lambda x}{\left[1-e^{-\lambda x}\right]}^{M_{\lambda }P-1}\right]f\left(P|\text{WPP}\;2019\;\text{Data}\right)f\left(\lambda |\text{IDL}\;\text{Data}\right)dPd\lambda .$$

Equation (6) is obtained from the previous line using equations (5) and (2). Since we do not have access to the analytic form of the posterior distribution of P, we instead approximate the unconditional density f(x) by Monte Carlo using the following simulation algorithm:


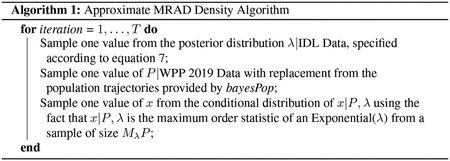


The collection of the sampled values of x from Algorithm 1 will approximate the unconditional distribution of MRAD this century.

The first step of the algorithm is based on the Bayesian posterior of λ∣X, where X denotes the sample of supercentenarian death ages from the IDL data. We note that our analysis must take into account the truncation present in the IDL, as discussed in Section 2.2. We denote by $C_{1}$ and $C_{2}$ the sets of individuals i∈{1,…,n} that exhibit case (1) or case (2) of truncation. Then, using the standard vague prior π(λ)∝1/λ (Elfessi and Reineke 2001), we see that,

(7) $$f\left(\lambda |X\right)\propto f\left(X|\lambda \right)\pi \left(\lambda \right)\;=\left(\overset{n}{\underset{i=1}{\prod }}f\left(X_{i}|\lambda \right)\right)\pi \left(\lambda \right)\;=\left(\underset{i\in C_{1}}{\prod }f\left(X_{i}|\lambda \right)\right)\left(\underset{i\in C_{2}}{\prod }f\left(X_{i}|\lambda \right)\right)\pi \left(\lambda \right)\;\propto \left(\underset{i\in C_{1}}{\prod }\frac{\lambda e^{-\lambda X_{i}}}{\left(1-e^{-\lambda \left(e_{c_{i}}-t_{i}\right)}\right)-\left(1-e^{-\lambda \left(b_{c_{i}}-t_{i}\right)}\right)}\right)\left(\underset{i\in C_{2}}{\prod }\frac{\lambda e^{-\lambda X_{i}}}{\left(1-e^{-\lambda \left(e_{c_{i}}-t_{i}\right)}\right)}\right)\left(\frac{1}{\lambda }\right)\;=\frac{\lambda^{n-1}e^{-\lambda \sum_{i=1}^{n}X_{i}}}{\prod_{i\in C_{1}}\left(e^{-\lambda \left(b_{c_{i}}-t_{i}\right)}-e^{-\lambda \left(e_{c_{i}}-t_{i}\right)}\right)\times \prod_{i\in C_{2}}\left(1-e^{-\lambda \left(e_{c_{i}}-t_{i}\right)}\right)}.$$

## 4. Results

In this section, we carry out each step of the methodology described at the beginning of Section 3. Step 1 was completed in Section 2 by confirming the exponential survival model for supercentenarians and specifying the density of the maximum order statistic for that model, conditional on the exponential rate parameter λ.

**Figure 4:**
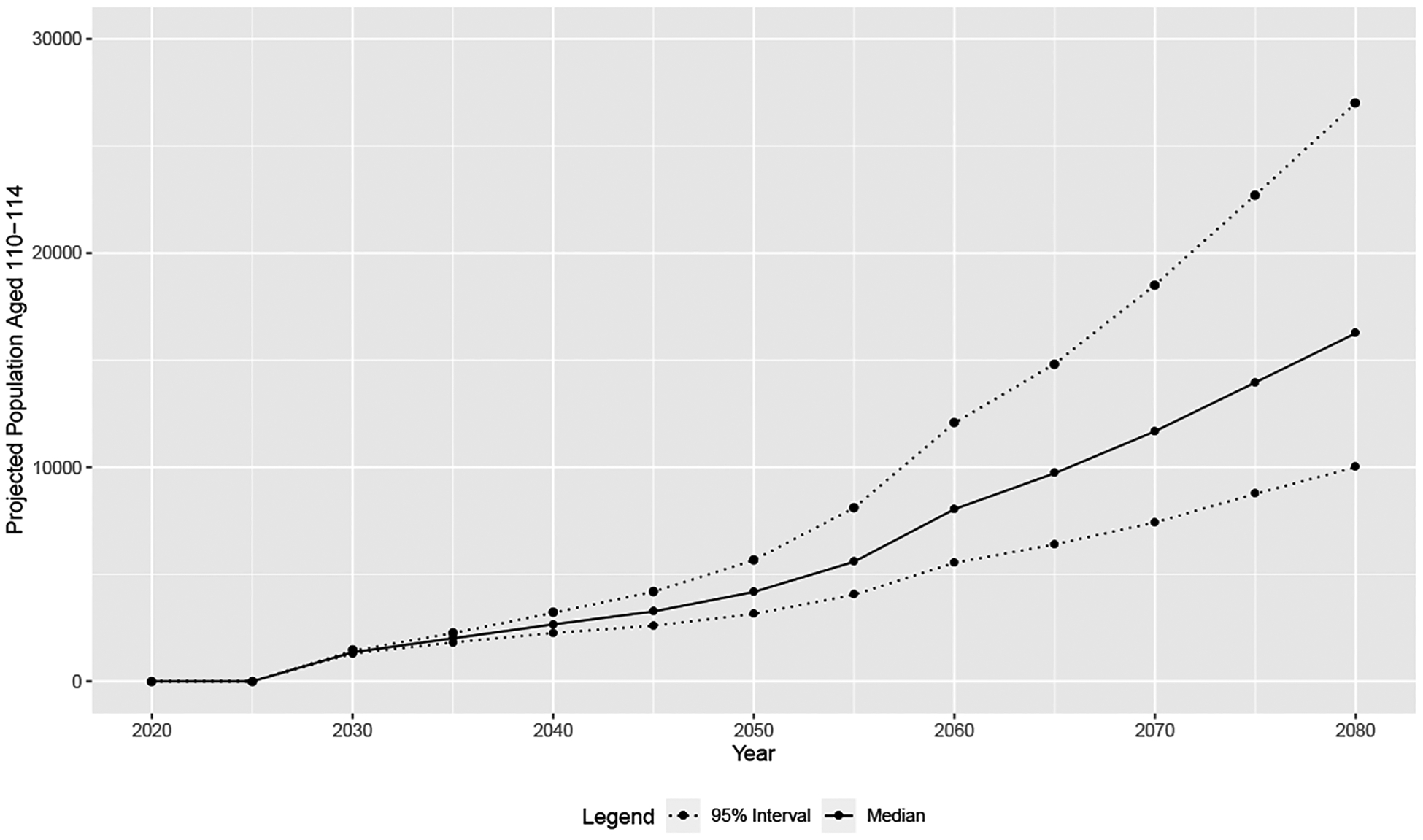
Median and 95% confidence interval population projections in age range 110–114 by year in 5-year increments

For Step 2, we obtain Bayesian population projections for the number of individuals to reach age 110 this century. Figure 4 shows median and 95% confidence intervals for population projections of ages 110–114 from 2020 to 2080 in five-year time intervals. We note that these are simply the projected populations in the age range 110–114 by year in 5-year increments, and do not account for the result from Proposition 1.

**Figure A-2:**
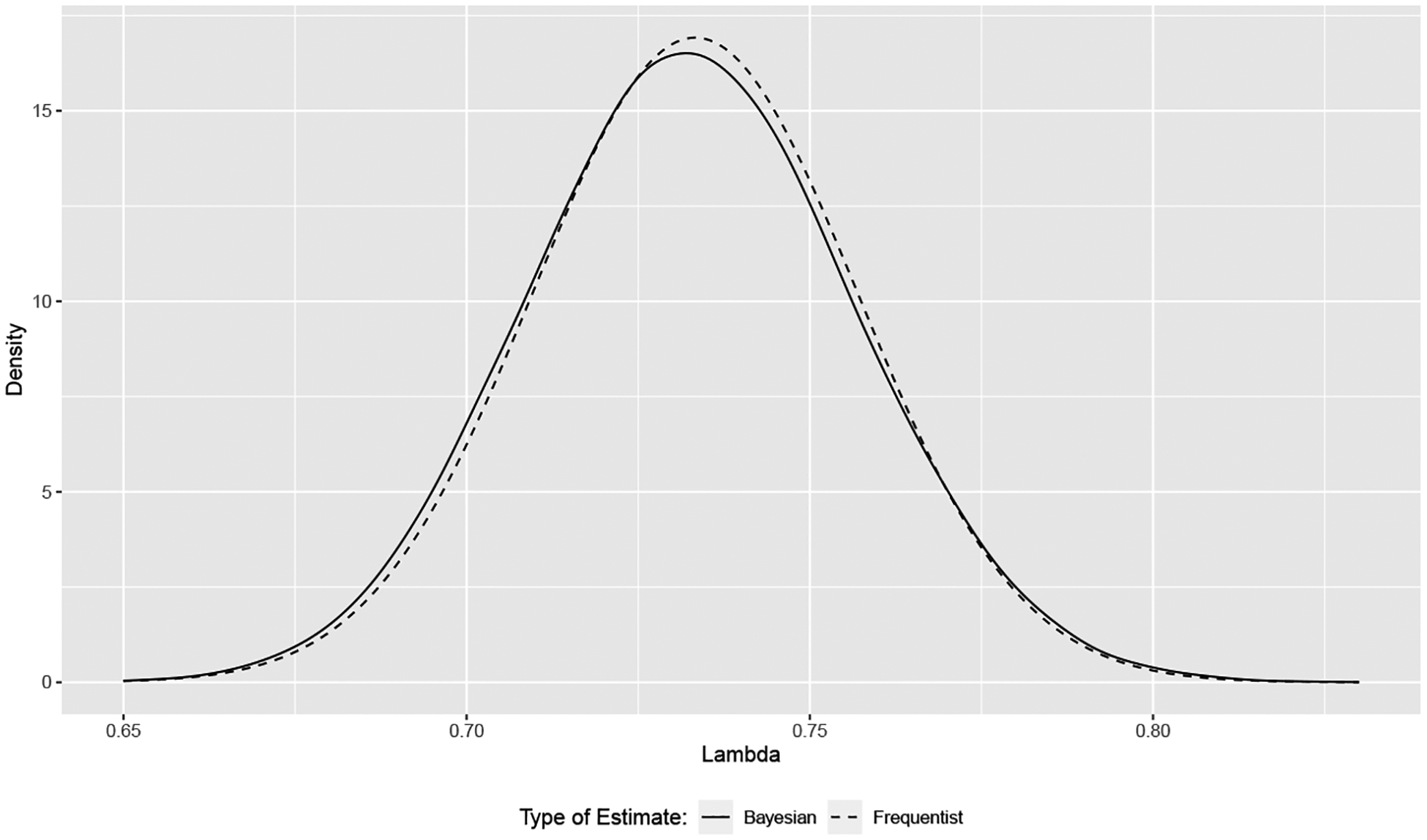
Posterior density for λ∣X

For Step 3, we first estimate the posterior distribution of λ∣X using equation (7) and a standard MCMC estimation procedure using the Metropolis-Hastings algorithm, as implemented in the *metrop* function of the *mcmc* package in R (Geyer and Johnson 2020). The estimated posterior distribution is shown in Appendix Figure A-2 using a simple kernel density estimate. We note that the distribution of $\overset{ˆ}{\lambda }$ from a frequentist analysis (as described in Section 2.2) would be $\overset{ˆ}{\lambda }\sim \text{Normal}\left(\mu =0.733,\sigma^{2}=0.0006\right.)$. This distribution is also plotted in Figure A-2 and is nearly identical to the estimated posterior from our Bayesian analysis.

**Figure 5:**
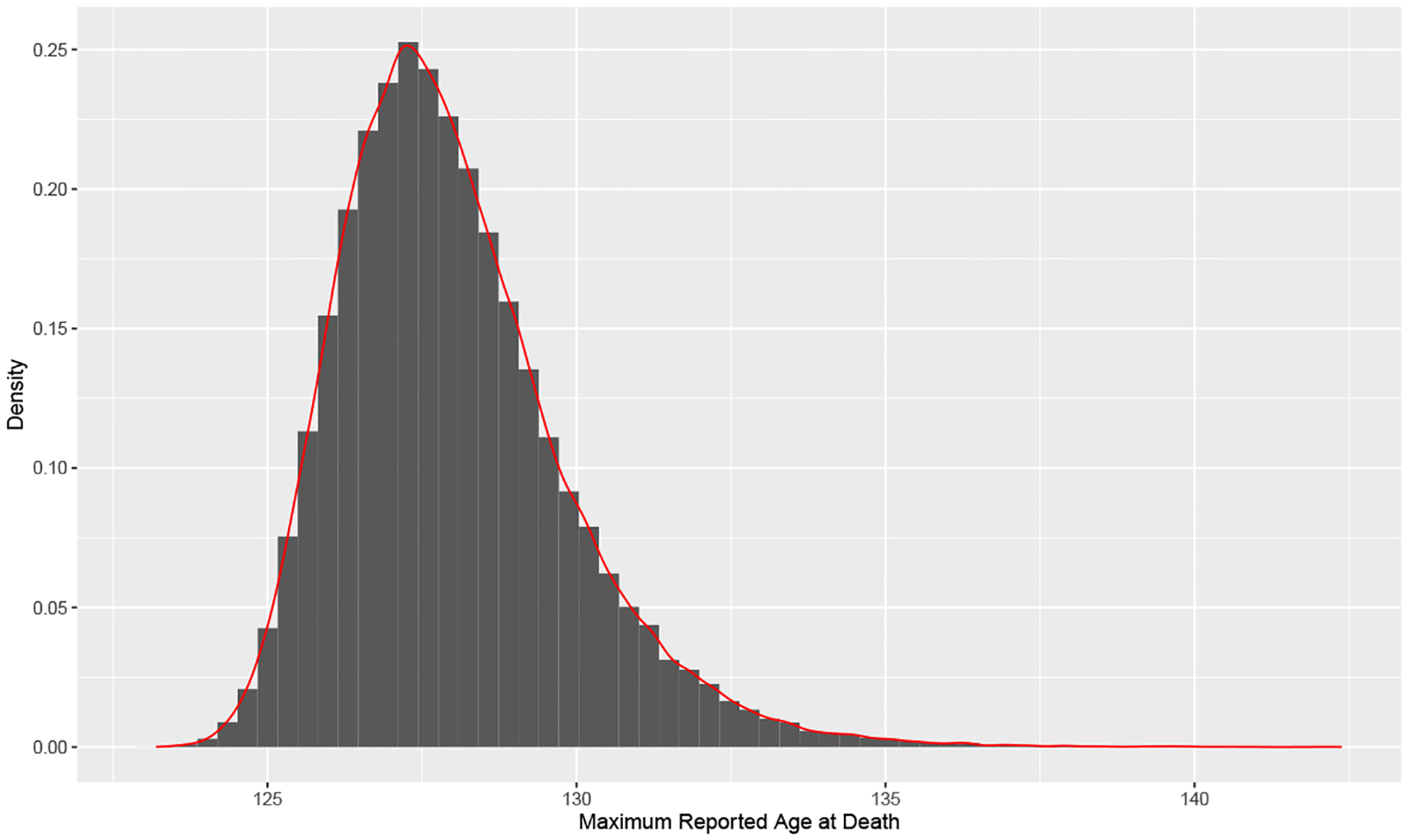
Posterior density histogram overlaid with approximate density (red) of MRAD in the period 2020–2100

Finally, we run Algorithm 1 to approximate the unconditional density of MRAD this century. We use $T=10^{5}$ iterations, which provided a smooth posterior distribution. The final results are shown in Figure 5, which includes both a histogram of sampled values and an estimated density using a simple kernel density estimate.

Table 2 displays the estimated probabilities that given ages are attained in the period 2020–2100, in terms of age at last birthday. For probabilities based on additional ages, refer to Appendix Table A-4.

## 5. Discussion

We have extended the work of Rootzén and Zholud (2017) by incorporating probabilistic projections of the number of individuals to reach age 110 this century and using the most recent IDL data to create the first unconditional probability density function of the maximum reported age at death (MRAD) this century with a fully Bayesian approach. Based on this methodology, we find that the probability of breaking the current MRAD record (122 years and 164 days, set by Jeanne Calment of France) this century is near 1, the probability of a person reaching age 126 is very high at approximately 89%, and the probability of a person reaching age 130 is still reasonable at nearly 13%. Although possible under the model, it is extremely unlikely that a person will attain age 135 or 140 this century.

Our results may be viewed as a way to resolve the apparent conflict between a limit to human lifespan and the lack of any specific bound to human life. The exponential survival model for supercentenarians suggests that the MRAD will continue to increase as more and more people reach age 110. However, the high year-over-year mortality rates implied by the model also suggest that the frequency at which these records are broken is likely to slow unless there are order of magnitude increases to the number of people to reach age 110. Under the most recent IDL data and population projections, it is quite likely that someone will reach age 125 this century, but not age 135.

There are a few details of the model to discuss. First, we note that technically our model applies only to supercentenarians who die on or after January 1, 2020, and not to any currently living supercentenarians. Ultimately, we believe their omission from our projections is (1) necessary due to data quality issues and (2) will not impact the results substantially. Regarding the first point, the available data from the IDL only includes supercentenarians who have already died. Although records of many alive individuals who reached age 110 by 2020 are quite possibly accurate, we do not have a unified and well-documented verification source that is free of age-attainment bias.

Regarding the second point, the key observation is that the number of supercentenarians alive on January 1st, 2020 is not sizable. As previously mentioned, order of magnitude increases to supercentenarian populations are necessary to meaningfully change the distribution of MRAD this century; the projections presented in Section 4 do not show any significant supercentenarian population until at least 2030. Furthermore, there are too few individuals alive today who are old enough to be likely to break the current MRAD. The current oldest living human is Kane Tanaka of Japan at age 118, with just three other individuals older than age 115. These records were verified by the Gerontology Research Group (GRG) which may not necessarily uphold the rigorous standards of the IDL (Gerontology Research Group 2021).

Also, the projections apply only to individuals from one of the 13 IDL countries listed in Section 2. Since the single-parameter exponential survival model for supercentenarians can be verified only for individuals from these countries, we have omitted individuals projected to reach age 110 from other countries from our analysis. This choice can only make our results more conservative as the probability that an individual from a IDL country becomes the record holder for MRAD this century is nonzero.

In any analysis of this type, it is important to emphasize the dependence of results on the accuracy of the supercentenarian survival data. Although the IDL provides an extremely rigorous verification of supercentenarians within country-specific periods, inaccuracies or missing data may bias the results. Records excluded from the IDL because they lack verification documents may in fact be “missing not at random” and bias the results up or down. Furthermore, there is always the possibility of age-attainment bias influencing the data, despite the best efforts of the IDL. As pointed out by Jdanov, Shkolnikov, and Gellers-Barkmann (2021), the enactment of new regulations will make the accurate collection and publication of supercentenarian records more challenging, since more and more records may be removed or excluded from the database as time progresses. If, for example, record-breaking individuals must be removed due to privacy protection laws, results will be biased low.

Regardless, as more and more people attain age 110 in the coming decades, it will be important to reverify the assumptions made in this analysis. Specifically, it will be necessary to confirm the flat mortality assumption and assumptions of no region- or sex-specific differences in mortality conditional on attaining age 110. If these assumptions do not continue to hold, our projection of the MRAD by 2100 may not either.

In summary, we have found that the probability an individual exceeds the current record for maximum reported age at death this century is near 1, although it is unlikely that any individual will live beyond age 135. Moreover, increases to the age record depend heavily upon substantial increases to the number of people to attain age 110 in the coming decades. Projected supercentenarian population increases suggest that ages previously argued to be impossible may soon be attained.

## Figures and Tables

**Table 1:** Likelihood ratio test (LRT) and BIC analysis of model assumptions

Test	Degrees of freedom	LRT statistic	p-value	BIC
Constant mortality (exponential vs. GP)	1	0.39	0.532	6.63
Region-specific mortality	3	6.74	0.081	14.32
Sex-specific mortality	1	0.42	0.518	6.60
Sex-specific mortality in North America	1	0.38	0.540	5.87
Sex-specific mortality in Northern Europe	1	0.63	0.426	4.75
Sex-specific mortality in Southern Europe	1	0.78	0.378	4.95
Sex-specific mortality in Japan	1	0.09	0.759	4.26

**Table 2:** Estimated unconditional probability of maximum reported age at death (MRAD) in the period 2020–2100

Age	Probability	Age	Probability
120	1.00000	132	0.03318
122	1.00000	134	0.00814
124	0.99938	136	0.00208
126	0.88777	138	0.00053
128	0.43703	140	0.00012
130	0.13223	142	0.00003

**Figure A-1:**
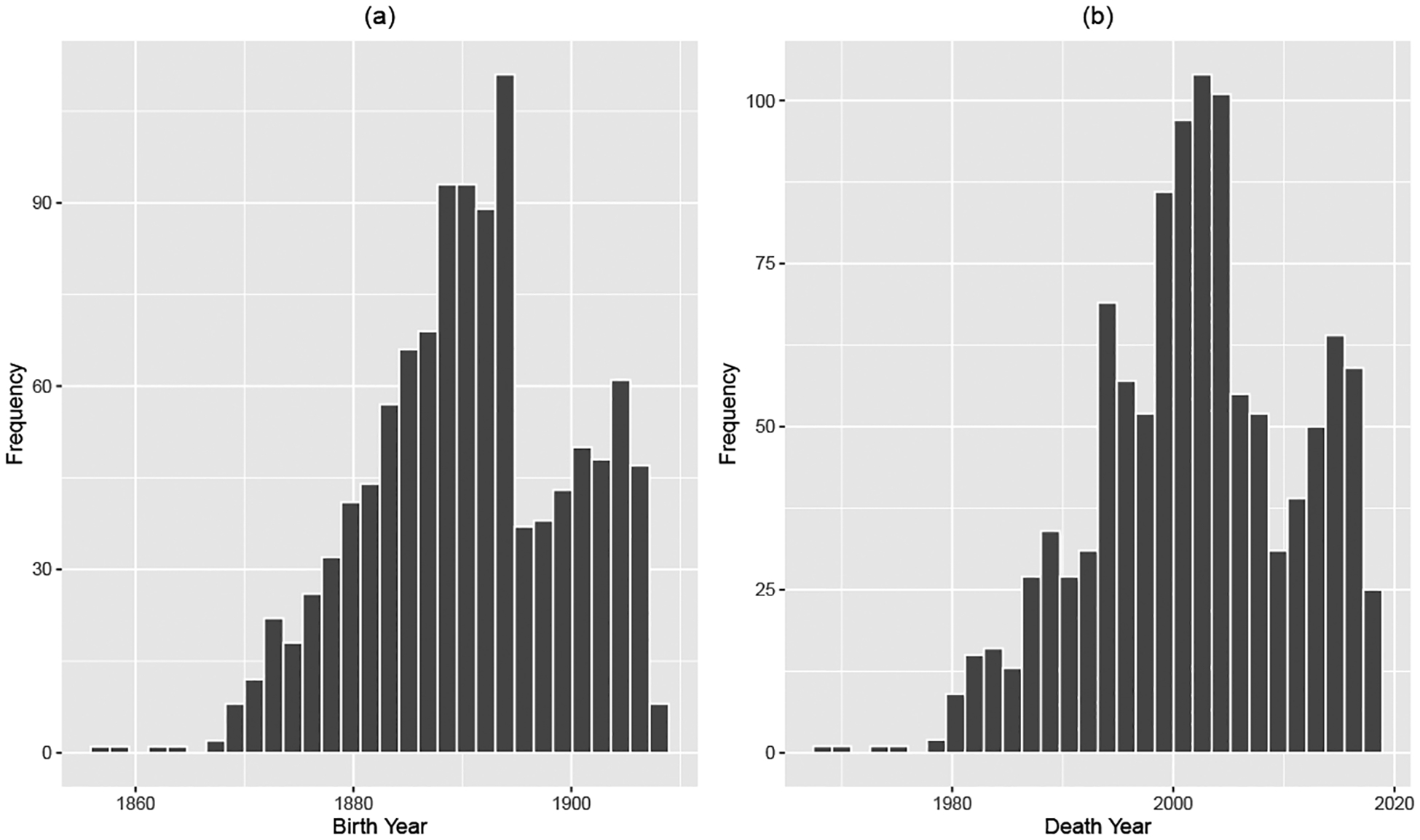
Histogram of birth (a) and death (b) years of supercentenarians in IDL v3

## Footnotes

3 The country of each individual is usually determined by place of death. We use the classifications stated in the metadata of the IDL website (Max Planck Institute for Demographic Research 2020).

## Appendix

### IDL v3 data summary

**Table A-1:** Counts of supercentenarians in IDL v3 by country

Country	Male	Female	Total
Austria	0	6	6
Belgium	2	17	19
Canada	0	12	12
Denmark	0	3	3
England and Wales	9	148	157
Finland	0	5	5
France	8	233	241
Germany	3	13	16
Japan	10	68	78
Norway	1	7	8
Spain	9	51	60
Sweden	1	9	10
USA	47	457	504
Totals	90	1029	1119

*Note*: Counts are by the country listed for each record in the IDL, which is usually place of death. Two individuals who died in Italy and Lebanon, respectively, are included in France’s totals as they were born in France.

**Table A-2:** Counts of supercentenarians by IDL update

v1	v2	v3
613	404	102

### Semisupercentenarian testing

In Table A-3 below, we conduct hypothesis testing via LRT and BIC to check for evidence of differences in mortality rates between semisupercentenarians and supercentenarians, as well as region-specific, sex-specific, and sex- and region-specific differences in mortality in an exponential model.

**Table A-3:** Semisupercentenarian and supercentenarian hypothesis testing results

Test	Degrees of freedom	LRT statistic	p-value	BIC
Difference in mortality (one versus two binomials)	1	104.109	<0.001	−94.490
Region-specific mortality	3	1190.657	<0.001	−1161.799
Sex-specific mortality	1	62.689	<0.001	−53.070
Sex-specific mortality, North America	1	81.510	<0.001	−74.441
Sex-specific mortality, Northern Europe	1	60.705	<0.001	−52.511
Sex-specific mortality, Southern Europe	1	13.678	<0.001	−4.450

*Note*: We do not test for sex-specific differences in mortality in Japan since there are no semisupercentenarians from that country in the IDL v3. The result would be the same as is presented in Table 1.

### Table of MRAD probabilities

**Table A-4:** Estimated unconditional probability of MRAD in the period 2020–2100

Age	Probability	Age	Probability
120	1.00000	132	0.03318
121	1.00000	133	0.01600
122	1.00000	134	0.00814
123	1.00000	135	0.00408
124	0.99938	136	0.00208
125	0.98324	137	0.00102
126	0.88777	138	0.00053
127	0.68112	139	0.00035
128	0.43703	140	0.00012
129	0.24813	141	0.00005
130	0.13223	142	0.00003
131	0.06739	143	0.00000
